# The dynamic range of circulating tumor DNA in metastatic breast cancer

**DOI:** 10.1186/s13058-014-0421-y

**Published:** 2014-08-09

**Authors:** Maryam Heidary, Martina Auer, Peter Ulz, Ellen Heitzer, Edgar Petru, Christin Gasch, Sabine Riethdorf, Oliver Mauermann, Ingrid Lafer, Gunda Pristauz, Sigurd Lax, Klaus Pantel, Jochen B Geigl, Michael R Speicher

**Affiliations:** 1 Institute of Human Genetics, Medical University of Graz, Harrachgasse 21/8, Graz, A-8010 Austria; 2 Department of Obstetrics and Gynecology, Medical University of Graz, Auenbruggerplatz 14, Graz, A-8036 Austria; 3 Institute of Tumor Biology, University Medical Center Hamburg Eppendorf, Martinistrasse 52, Hamburg, D-20246 Germany; 4 Department of Pathology, General Hospital Graz West, Goestingerstrasse 22, Graz, A-8020 Austria

## Abstract

**Introduction:**

The management of metastatic breast cancer needs improvement. As clinical evaluation is not very accurate in determining the progression of disease, the analysis of circulating tumor DNA (ctDNA) has evolved to a promising noninvasive marker of disease evolution. Indeed, ctDNA was reported to represent a highly sensitive biomarker of metastatic cancer disease directly reflecting tumor burden and dynamics. However, at present little is known about the dynamic range of ctDNA in patients with metastatic breast cancer.

**Methods:**

In this study, 74 plasma DNA samples from 58 patients with metastasized breast cancer were analyzed with a microfluidic device to determine the plasma DNA size distribution and copy number changes in the plasma were identified by whole-genome sequencing (plasma-Seq). Furthermore, in an index patient we conducted whole-genome, exome, or targeted deep sequencing of the primary tumor, metastases, and circulating tumor cells (CTCs). Deep sequencing was done to accurately determine the allele fraction (AFs) of mutated DNA fragments.

**Results:**

Although all patients had metastatic disease, plasma analyses demonstrated highly variable AFs of mutant fragments. We analyzed an index patient with more than 100,000 CTCs in detail. We first conducted whole-genome, exome, or targeted deep sequencing of four different regions from the primary tumor and three metastatic lymph node regions, which enabled us to establish the phylogenetic relationships of these lesions, which were consistent with a genetically homogeneous cancer. Subsequent analyses of 551 CTCs confirmed the genetically homogeneous cancer in three serial blood analyses. However, the AFs of ctDNA were only 2% to 3% in each analysis, neither reflecting the tumor burden nor the dynamics of this progressive disease. These results together with high-resolution plasma DNA fragment sizing suggested that differences in phagocytosis and DNA degradation mechanisms likely explain the variable occurrence of mutated DNA fragments in the blood of patients with cancer.

**Conclusions:**

The dynamic range of ctDNA varies substantially in patients with metastatic breast cancer. This has important implications for the use of ctDNA as a predictive and prognostic biomarker.

**Electronic supplementary material:**

The online version of this article (doi:10.1186/s13058-014-0421-y) contains supplementary material, which is available to authorized users.

## Introduction

Measuring treatment response in patients with metastatic breast cancer is usually done by serial clinical evaluation of symptoms and estimates of tumor burden. However, serial radiographic imaging is expensive, often inconclusive, and may fail in detecting changes in tumor burden. Cancer antigen 15-3 (CA 15-3) has reasonable sensitivity, but changes in levels do not necessarily reflect tumor response or progression [1]. Hence, `liquid biopsies', that is, analyses of circulating tumor cells (CTCs) or plasma DNA, have recently acquired considerable interest [2]-[4]. Indeed, the enumeration of CTCs has evolved to a promising biomarker [5]. The CellSearch System (Janssen Diagnostics, LLC, New Brunswick, NJ, USA) has been cleared by the Food and Drug Administration, as increased CTC numbers, that is five or more cells per 7.5 ml of blood, in patients with metastatic breast cancer have been associated with a worse prognosis [6],[7]. As a substantial fraction of patients with metastatic cancer have unexpectedly low CTC counts when assessed with this system, new CTC assays are under development [3].

Furthermore, tumor cells release DNA fragments into the circulation, termed circulating tumor DNA (ctDNA), which can be found in the cell-free fraction of blood together with DNA fragments from normal cells, which is commonly referred to as cell-free DNA (cfDNA) [3],[8],[9]. Tumor-specific sequence alterations in plasma were used to quantify tumor burden [10]-[13] or for genome-wide analyses of tumor genomes [14]-[19]. Multiple studies have suggested that ctDNA can be used to monitor tumor dynamics [10]-[14],[16],[17],[20]-[23]. For example, a recent study has reported that in women with breast cancer the ctDNA levels showed a greater dynamic range, and greater correlation with changes in tumor burden than CA 15-3 or CTCs [13]. For these reasons, it was even proposed that the serial analysis of cancer genomes in plasma constitutes a new paradigm for the study of clonal evolution in human cancers [17].

We studied ctDNA in 58 women with metastasized breast cancer. We exemplify our observations using an index case with extensive metastases to the bones and liver and excessive CTC numbers (*n* = approximately 50,000 to >100,000) in serial analyses, which we examined with whole-genome, exome and targeted deep sequencing.

## Methods

### Plasma DNA extraction and sizing

Plasma DNA was extracted as previously described [15]. The size distribution of plasma DNA fragments was evaluated on an Agilent 2100 Bioanalyzer using the DNA series Agilent High Sensitivity DNA kit (Agilent Technologies, Santa Clara, CA, USA).

### Collection and procession of material from the index patient

**Figure 1:**
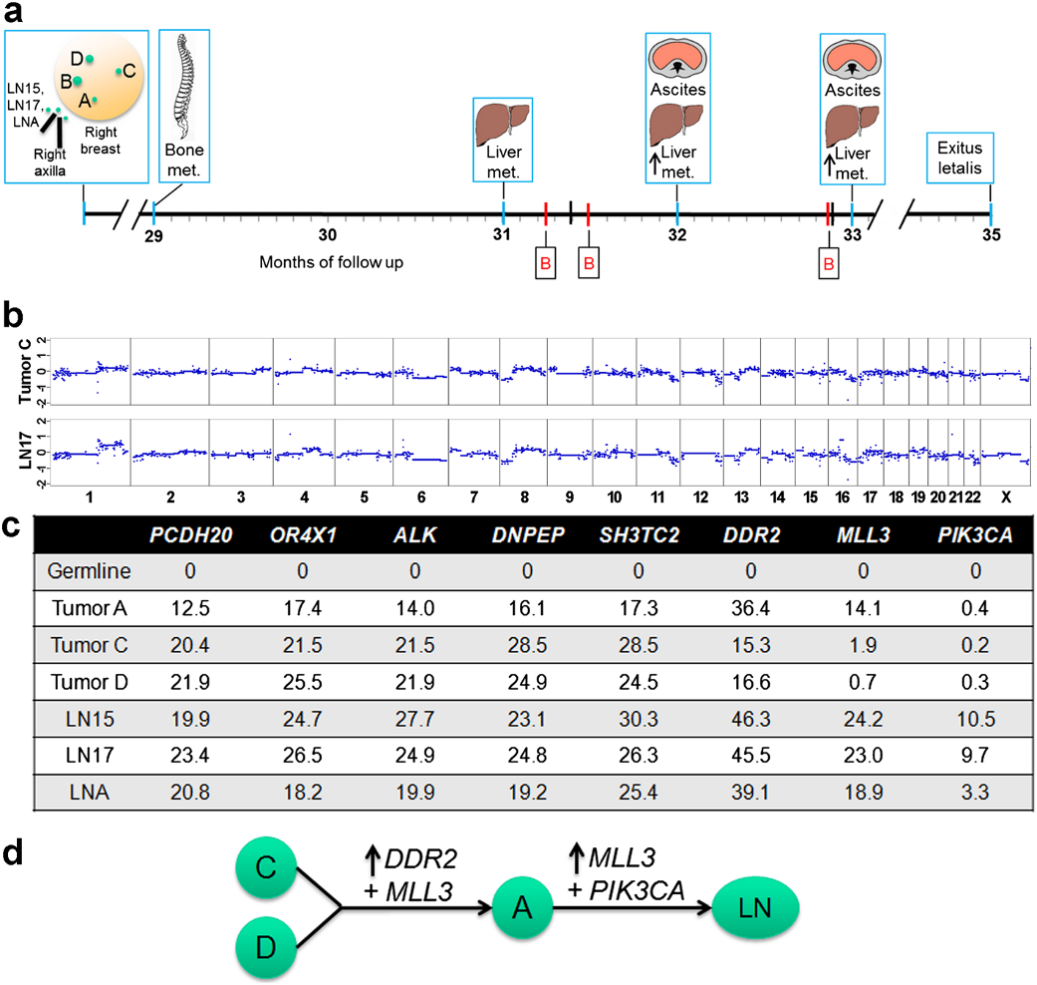
**Timeline of the clinical course of the index patient and results obtained from the analyzed primary tumor and metastatic lesions. (a)** The timeline starts with a sketch illustrating the localization of the lesions we subjected to our analyses, that is primary tumor lesions A, B, C, and D and lymph node metastases LN15, LN17, and LNA from the right axilla as observed at the time of diagnosis. In the timeline, dates when clinical progress was noted are indicated with a blue bar and the time points of blood collections (B) by a red bar (for details see text). **(b)** Copy number profiles obtained by whole-genome sequencing from tumor C and lymph node metastasis LN17. The X-axis shows the chromosome, the Y-axis indicates log_2_-ratios. **(c)** Mutation frequencies of genes *PCDH20*, *OR4X1*, *ALK*, *DNPEP*, *SH3TC2*, *DDR2*, *MLL3*, and *PIK3CA* as established by targeted deep sequencing in the tumor lesions and lymph node metastases. **(d)** The clonal evolutionary relationships of the analyzed lesions based on the frequency of single nucleotide variants (SNVs) as shown in (c). The arrow indicates an increase in the frequency of the respective SNV, the `+' a newly occurred SNV. The three lymph node metastases are indicated together in the circle labeled `LN'.

Material was obtained by microdissection from the four largest tumor foci, designated as tumor A (diameter: 6 mm), B (12 mm), C (8 mm), and D (11 mm), and from five metastatically involved lymph nodes, designated as LN15 (one lymph node metastasis of 1 cm diameter), LN17 (three neighboring lymph node metastases of 3, 6 and 8 cm, respectively, in diameter, embedded together in one block and harvested together for DNA extraction), and LNA (a sentinel node with 1.2 cm metastasis) (Figure 1). From all lesions a tumor DNA content of at least 70% was acquired. In addition, we obtained saliva in order to get tumor-free, germline DNA. We collected blood for CTC and ctDNA analysis at three different time points (Figure 1). From these blood samples we prepared plasma DNA and enumerated CTCs using the FDA-cleared CellSearch system (Janssen Diagnostics, LLC). For further analyses, CTCs were isolated from the CellSearch system as previously described [24]. In addition, we isolated CTCs after Oncoquick density gradient centrifugation and staining with EpCAM and CD45 using the CellCelector (ALS, Jena, Germany), based on their staining pattern, that is EpCAM+/CD45- cells. DNA from CTCs was subjected to whole-genome amplification (WGA), that is lysis with protease followed by Phi29 amplification as described [24].

All blood samples were collected before the administration of each treatment cycle. The study was approved by the ethics committee of the Medical University of Graz (approval number 21-227 ex 09/10) and conducted according to the Declaration of Helsinki, written informed consent was obtained from all patients. The index patient has provided informed consent to publish the information contained in this manuscript.

### Isolation of CTCs using the CellSearch or the CellCelector systems

Blood samples (7.5 ml each) were collected into CellSave tubes (Veridex, Raritan, NJ, USA). We have described capturing of CTCs with the CellSearch system before [24],[25]. In brief, CTCs were enriched and enumerated employing the Epithelial Cell Kit (Veridex). Subsequently, we captured CTCs by anti-epithelial cell adhesion molecule (EpCAM)-antibody-bearing ferrofluid. We identified CTCs based on cytokeratin-positivity and negativity for the leukocyte common antigen CD45. Furthermore, cells were stained with 4',6-diamidino-2-phenylindole (DAPI) to evaluate the integrity of the nucleus.

For the CellCelector system (ALS) we first performed density gradient centrifugation with Oncoquick™ (Greiner Bio-One, Kremsmunster, Austria) and then cells were stained with anti-EpCAM and anti-CD 45 antibody. Using the CellCelector, only EpCAM-positive and CD45-negative cells were isolated with a 50 μm capillary at an amount of 0.8 μl phosphate-buffered saline and transferred into the cap of a 200 μl tube, containing 7 μl of nuclease-free water. After centrifugation cells were frozen for at least one hour at -20°C prior to WGA.

### Whole-genome amplification for CTC analysis

The DNA of single or few CTCs had to be subjected to WGA for further analysis. The WGA protocol was described in detail in our previous publications [24],[26]-[28].

### Establishment of copy number profiles

In order to establish copy number profiles from the various samples we prepared libraries and subjected them to whole-genome sequencing at a shallow sequencing depth (0.1x) as previously described [18],[19]. For CTC analyses, we used - after WGA - either whole-genome sequencing or array-CGH to determine copy number changes. The array-CGH protocols were published previously [24],[26]-[28].

### Exome sequencing; somatic mutations

For all primary tumor lesions (A, B, C, and D), for lymph node metastases (LN15, LN17, and LNA), and for the constitutional DNA from saliva we sequenced the complete coding regions of the genome, that is conducted exome sequencing at a sequencing depth of approximately 50x. Prior to exome enrichment 300 to 400 bps libraries were prepared using the TruSeq DNA Sample Prep Kit (Illumina, San Diego, CA, USA). Exome enrichment was performed using the TruSeq Exome Enrichment Kit (Illumina) according to the manufacturer's instructions. Briefly, libraries were pooled equimolarily and hybridized in two reactions to the exome capture probes at 58°C for 16 to 20 h. Streptavidin beads were used to pull down the complex of captured oligos and genomic DNA fragments, whereas unbound fragments were removed by a first wash. To further enrich the target regions, a second hybridization was performed followed by another pull down with streptavidin beads. Finally, the enriched fragment pools were amplified for 15°Cycles of PCR. Enriched libraries were quality checked on an Agilent 7500 DNA kit (Agilent Technologies) and quantified using qPCR. The enriched library pools were then sequenced across two lanes of a HiSeq 2000 (Illumina), where a mean of 49 million reads for the formalin-fixed paraffin-embedded samples and 153 million reads for the constitutional DNA was obtained.

### Bioinformatics

Sequences were aligned to the human genome (hg19) using Burrows-Wheeler Algorithm (BWA; MEM-algorithm, version 0.7.4-r385) [29] with default alignment parameters. Somatic single nucleotide polymorphisms (SNPs) were called using the MuTect algorithm [30]. In addition, we generated constitutional SNP calls from exome reads of the constitutional DNA by using the UnifiedGenotyper of the Genome Analysis Toolkit (version 1.6-13) [31].

In order to identify phylogenetic relationships between copy number profiles of tumors, lymph nodes, and CTCs, we calculated the distance matrix (from Manhattan distances) based on the copy number status of the 50,000 bins from the whole-genome sequencing approach and subsequently created a dendrogram. To summarize copy number aberrations of unbalanced plasma samples, we subjected our plasma-Seq data to the Genomic Recurrent Event ViEwer (GREVE) algorithm [32]. Details on settings of the software parameters are available on request.

### Variant prioritizing

We utilized exome sequencing from DNA obtained from saliva (germline DNA), tumor lesions A, C, D (the DNA quality from tumor B was not sufficient for exome sequencing) and for the metastases LN15, LN17, and LNA to search for single nucleotide variants (SNVs) informative for establishing a phylogenetic relationship.

After applying the aforementioned filter and restriction of analysis to exons with their adjacent 2 bps on each side, exome sequencing identified 21,741 SNVs in the germline DNA, 20,749 of which were in the Single Nucleotide Polymorphism Database (dbSNP132) and 992 were novel. We then used the exome data from the tumor lesions to filter for somatic SNVs and found somatic SNV numbers between 716 (tumor C) and 1,589 (LN15). In order to minimize our dataset and therefore make it feasible for validation of the identified variants with an independent method, we first focused on mutations in known driver genes that are listed in the Catalogue of Somatic Mutations (COSMIC; [33]), thereby reducing the number of SNVs to <20/sample.

Subsequently, we filtered for identical SNVs present in more than one sample and confirmed those SNVs by targeted amplicon sequencing. These efforts resulted in the identification of SNVs in eight genes (that is *PCDH20*, *OR4X1*, *ALK*, *DNPEP*, *SH3TC2*, *DDR2*, *MLL3*, and *PIK3CA*) (Table S1 in Additional file 1), which appeared to be informative for addressing the clonal relationship of the various lesions. We then used targeted deep sequencing to accurately establish the allele frequency of these SNVs in each sample.

### Targeted deep sequencing

We used somatic mutations as established in the previous steps to conduct targeted deep sequencing of tumor samples, CTC amplification products, and plasma DNA. For the latter DNA samples deep sequencing of PCR products allowed us to determine the allele fraction (AF) of the mutant alleles in the sample analyzed. Targeted deep sequencing was performed on the Illumina MiSeq™ System. In brief, primers were designed for all mutations as listed in Table S1 in Additional file 1 and Illumina-specific adapters were attached to the 5' ends in round of PCR. PCR was performed using the FastStart High Fidelity PCR System (Roche, Basel, Switzerland) according to the manufacturers' recommendation. The PCR products were purified using the Agencourt AMPure kit (Beckman Coulter, Brea, CA, USA) as described in the manufacturer's protocol and purified products were quantified using a Bioanalyzer High Sensitivity Chip (Agilent Technologies). The PCR products were pooled equimolarly and sequenced on the Illumina MiSeq™ System in a 2 × 150 bps run. Obtained sequence reads were base called, filtered by quality metrics, and aligned to the human reference sequence. We set a cutoff of 1% mutated fragments for reliable detection of mutant alleles in plasma DNA.

### Accession numbers

All sequencing raw data were deposited at the European Genome-phenome Archive (EGA, [34]), which is hosted by the EBI, under the study accession number EGAS00001000625.

## Results and discussion

Our aim was to investigate the significance of mutant AFs in a series of plasma samples from patients with metastatic breast cancer. As there is an ongoing discussion whether ctDNA and CTCs are rivals or partners in cancer care [9], we first describe our comprehensive analyses of an index patient with more than 100,000 CTCs in serial analyses and provide in parallel detailed single CTC and ctDNA analyses. In order to explore to what extent the ctDNA findings from this index patient are generalizable, we then extended our analyses by collecting mutant AFs in a further 71 plasma samples from 57 patients with metastasized breast cancer.

### History of the index case with more than 100,000 CTCs

The index case was a 40-year-old premenopausal woman diagnosed with a right-sided multifocal (seven lesions), invasive lobular breast cancer. The location of four of the seven foci which we analyzed is illustrated in Figure 1a. They were estrogen (ER) and progesterone (PR) receptor positive (Allred score of 8), without Her2/neu overexpression and amplification and a low Ki-67 labeling index of 5% to10%. After surgery, tumor stage was pT-1c (7), G-2, pN-3a (14/20). After adjuvant cytotoxic, epirubicin and cyclophosphamide chemotherapy she received anti-hormonal therapy with tamoxifen. There was no evidence of disease for two years and five months. However, 29 months after initial diagnosis, bone metastases and a thrombocytopenia (59,000/mm^3^) were noted (Figure 1a). Chemotherapeutic options were discussed with the patient but she refused. Therefore, the only treatment during our study period consisted of various endocrine regimens (for more details see Additional file 2). At month 31 of follow-up, several liver metastases were noted and we obtained our first and second blood samples about one and two weeks later, respectively (Figure 1a). Both blood samples yielded the exceptional number of more than 100,000 CTCs with the CellSearch system. At month 32 an increase of the liver metastases was noted and furthermore ascites were newly diagnosed. The physical condition of the patient (Karnofsky 60) worsened significantly over time and about one month later, we obtained the third blood sample (Figure 1a), which revealed a CTC number of approximately 50,000. White blood cell counts were with 9.02 G/l, 5.7 G/l, and 8.95 G/l at the first, second and third blood collections, respectively, within the normal range, no progenitor cells were observed. Further clinical signs of subileus were noted and the patient died about nine weeks after our last blood collection.

### Primary tumor lesions and lymph nodes were genetically homogeneous

We first addressed the question whether the spatially separated foci (the four analyzed lesions were designated as A to D; Figure 1a) from the primary tumor were clonally related. Copy number analyses performed by whole-genome sequencing identified marked similarities characterized by common gains and losses in these separated foci (that is gains of 1q and 8q, and losses of 6q, 8p, distal 11q, and 17p), suggesting a common origin of these lesions (the copy number profile of tumor C is exemplarily illustrated in Figure 1b; see Figure S1a in Additional file 3 for all profiles). We next applied whole-genome sequencing to three lymph nodes (LN15, LN17, and LNA), which again revealed similar copy number changes as the lesions from the primary tumor (Figure 1b, and Figure S1b in Additional file 3). Hence, copy number analyses were suggestive of a close clonal relationship between the various lesions.

To evaluate this further we utilized exome sequencing for DNA obtained from saliva (germline DNA) and the aforementioned lesions. After variant prioritizing (see Methods), we focused on SNVs in genes from the COSMIC database [33] that were present in three or more samples. This resulted in the identification of SNVs in eight genes (that is *ALK, DNPEP, OR4X1, PCDH20, SH3TC2*, *DDR2*, *MLL3*, and *PIK3CA*) (Table S1 in Additional file 1), which appeared to be informative for addressing the clonal relationship of the various lesions. We then used targeted deep sequencing to establish the AF of these SNVs accurately in each sample with the exception of tumor B, for which there was not enough DNA left. Read counts of mutated and total reads are listed in Table S1 in Additional file 1.

These efforts indeed confirmed the monoclonal origin of the various tumor lesions, because somatic SNVs in five genes (that is *ALK, DNPEP, OR4X1, PCDH20*, and *SH3TC2*) were present in all lesions with approximately the same AF (Figure 1c). The three other genes (that is *DDR2*, *MLL3*, and *PIK3CA*) allowed us to gain some insight into the clonal evolutionary relationships of the analyzed lesions. The SNV in the *DDR2* gene in tumor A had an increased AF (36.4%) as compared to tumors C (15.3%) or D (16.6%) and, furthermore, tumor A had a SNV in *MLL3*, which was not observed in tumors C and D. This *MLL3* SNV was also observed in the three lymph nodes, each with a higher AF (from 18.9% in LNA to 24.2% in LN15) than in tumor A (14.1%). In addition, the lymph nodes had a novel SNV in the *PIK3CA* gene, which had not been present in tumors A, C, or D. This SNV was the N345K mutant, which was previously reported to change the inactive cytosolic confirmation of *PIK3CA* to an activated form on membranes [35]. Together, these genetic characteristics suggested that tumors C and D were common ancestors to the other lesions, tumor A had descended from tumor C and/or D, and the three lymph nodes had descended from tumor A (Figure 1d).

### CTC analyses revealed high degree of similarities to the primary tumor and metastases

**Figure 2:**
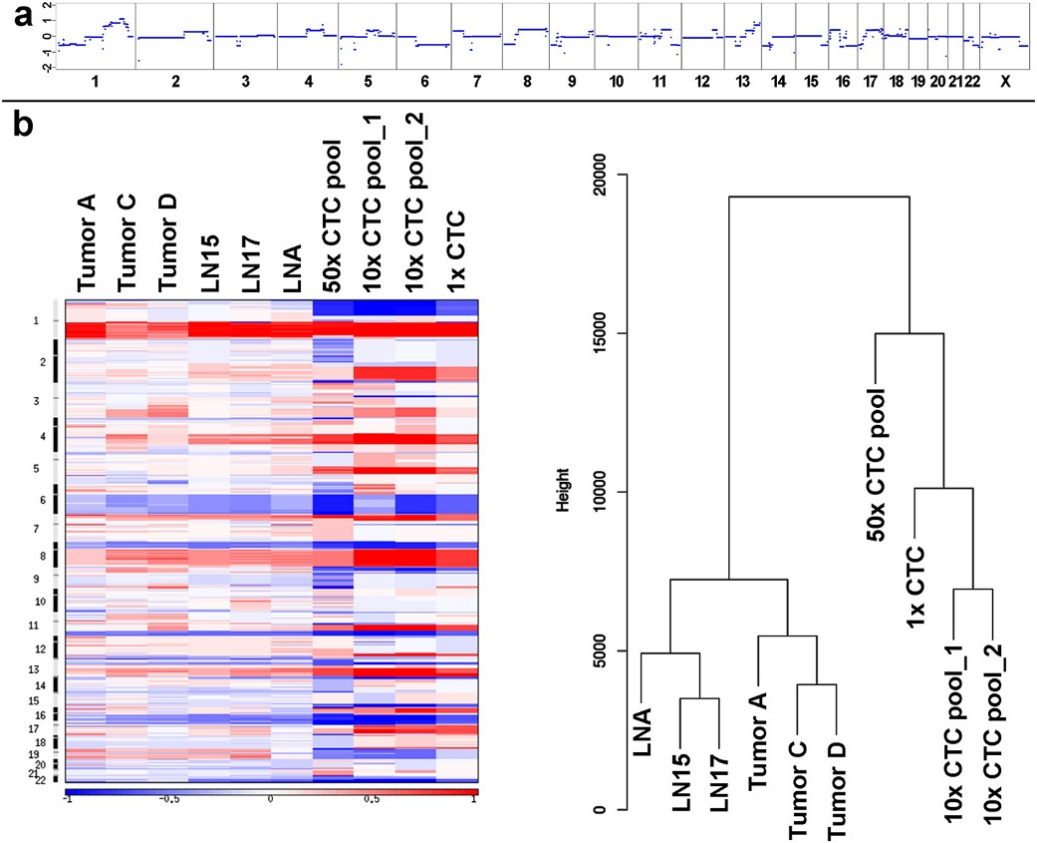
**Analyses of circulating tumor cells (CTCs) from the index patient. (a)** Whole-genome sequencing profile from a single CTC. **(b)** Left panel: heat maps from the copy number changes (red: overrepresentation, blue: underrepresentation) demonstrating the similarities between the various analyzed samples. Right panel: hierarchical clustering (Manhattan distance, complete agglomeration), the dendrogram illustrates the clonal relationships.

Between initial diagnosis and our first blood collection 31 months had passed. Hence, an important question was whether the tumor genome had evolutionarily adapted and developed novel changes. The CellSearch system (Janssen Diagnostics, LLC) revealed an extraordinary count of more than 100,000 CTCs at the first two blood collections and more than 50,000 CTCs at the third. Hence, these CTCs provided an extraordinary opportunity to address the aforementioned questions. In order to subject these CTCs to our single cell analysis tools [24],[27],[28],[36], we isolated them employing the CellSearch (Janssen Diagnostics, LLC) or the CellCelector (ALS) systems. Altogether we successfully analyzed 551 CTCs, either as single cells (*n* = 31) or in pools with various cell numbers (that is 5x = 6; 10x = 6; 25-50x = 5; 50x = 3; 100x = 1). Indeed, our CTC analyses revealed at all three time points similar patterns of copy number changes (that is again gains of 1q and 8q, and losses of 6q, 8p, distal 11q, and 17p) (an exemplary whole-genome sequencing profile of a single CTC is illustrated in Figure 2a; further whole-genome sequencing profiles are depicted in Figure S1c in Additional file 3 and array-CGH profiles in Figure S2a-b in Additional file 4). As a novel change in the CTCs we observed loss of chromosome 1p. We generated heat maps to illustrate the high degree of similarities of copy number changes among all analyzed tumor samples (Figure 2b).

Furthermore, we used deep sequencing to test the WGA products of the CTCs for the presence of the SNVs in the aforementioned eight genes. We detected the SNVs from all eight genes in all CTC WGA products, especially the *MLL3* and *PIK3CA* SNVs (Table S1 in Additional file 1), suggesting that the CTCs descended from the clones forming the lymph node deposits. When we used the copy number data for hierarchical clustering, the obtained dendrogram reflected the clonal relationships as established by the SNV patterns, that is tumors C and D clustered together with some distance to tumor A, whereas the lymph node deposits and the CTCs formed clusters of their own (Figure 2b).

### ctDNA had unexpected low allele frequencies

**Figure 3:**
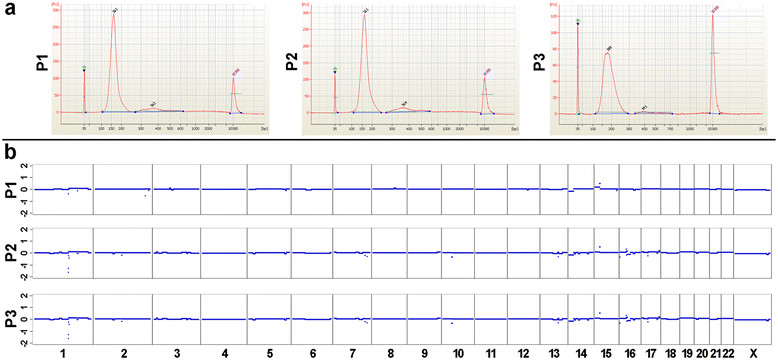
**Plasma DNA analyses from the index patient. (a)** Sizing of the plasma DNA fragments with a microfluidic device, that is the Agilent 2100 Bioanalyzer. All three plasma samples from the index patient (P1, P2, and P3, which correspond to the first, second, and third blood sample) demonstrated enrichment of short DNA fragments (size range 85 to 250 bps), whereas longer DNA size fragments (250 to 450 bps) were barely present. **(b)** Whole-genome sequencing (plasma-Seq) profiles of the three blood samples.

Due to the extensive metastatic and progressive disease, the presence of similar copy number changes, and identical mutations in all analyzed tumor deposits, we expected to detect tumor-associated changes in plasma DNA with ease, as ctDNA is supposed to reflect tumor burden [10]-[13],[21],[37]. As described previously [15], we first used a microfluidics-based platform for sizing of plasma DNA fragments and observed in all three measurements (P1, P2, and P3) an enrichment of plasma DNA fragments within the range of 85 to 250 bps, but not of DNA fragments with longer sizes, that is in the range of 250 to 450 bps (Figure 3a). This was unexpected, because we had previously reported that the presence of such longer size DNA fragments indicates a high frequency of ctDNA in plasma [15]. Indeed, when we performed whole-genome sequencing (plasma-Seq) [18],[19] we obtained balanced copy number profiles from all three samples (Figure 3b). As the fraction of tumor DNA in plasma can modulate copy number aberrations we then used the aforementioned eight somatic mutations to determine the mutant AF with targeted deep sequencing. A mean mutant AF of 3.7% ± 1.6% confirmed the low amount of tumor DNA in all plasma samples (Table S1 in Additional file 1).

We used the deep-sequencing data to calculate the number of mutant DNA fragments circulating in the index patient. By deep sequencing we found for the eight analyzed mutations on average 6,320, 8,524, and 4,419 mutant fragments in the first, second and third blood collection, respectively (Table S1 in Additional file 1). Based on previously published calculations, which assumed that the volume of distribution of DNA at steady state is similar to that of oligonucleotides in primates (60 to 70 ml/kg) [37], these mutant molecule numbers would correspond to 2.3*10^7^, 3.1*10^7^, and 1.6*10^7^, respectively, mutant fragments in total blood (for an approximate body weight of 60 kg for the index patient) at the time points of the three blood collections. As the half-life of ctDNA is short and was estimated at 16 min [38], the number of mutant molecules should approximately correspond to the number of tumor cells that released ctDNA at the time of the blood collections. Based on these calculations the number of mutated DNA fragments appears to be high. However, we had previously found mutant AFs in patients with metastatic colorectal or prostate cancer exceeding 30% [15],[18]. Murtaza *et al*. reported patients with metastasized breast cancer where plasma samples had mutant AFs exceeding 50% [17]. Considering the extensively metastasized disease and the exceptional number of CTCs, the low mutant AF in the index patient was unexpected. Our whole-genome, exome, and deep-sequencing data of primary tumor lesions, metastases, and altogether 551 CTCs indicated a common origin and presence of a genetically homogeneous cancer in this patient. Hence, it is unlikely that tumor heterogeneity or dominance of a clone with different characteristics than those of the analyzed regions may have obscured our analyses.

### Mutant AFs in plasma of patients with metastatic breast cancer are highly variable

As shown in the index case accurate quantification of ctDNA, AFs can be achieved by targeted deep sequencing. However, the comprehensive catalogs of publically available genomic breast cancer data generated by the Cancer Genome Atlas project revealed that only few genes are recurrently mutated in breast cancer [39]. Instead of massively parallel sequencing of primary tumors to identify somatic mutations suitable for AF ascertainment an easier approach is plasma-Seq, as it is independent of pre-knowledge of somatic mutations. We had previously shown [18],[19] that plasma-Seq detects tumor-specific copy number aberrations in plasma if the mutant AF exceeds 10%, whereas lower mutant AFs may appear as balanced profiles as in the index case.

**Figure 4:**
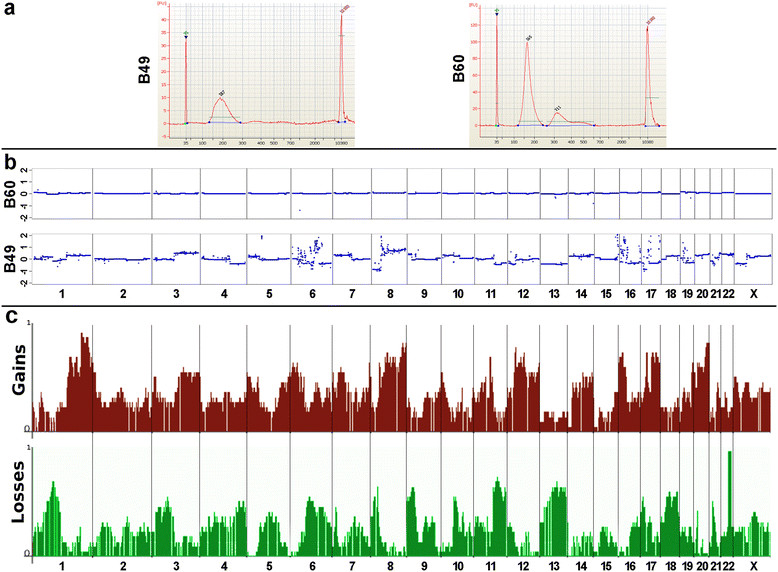
**Exemplary plasma DNA analyses from patients B49 and B60 and plasma DNA copy number patterns from a panel of patients with metastatic breast cancer. (a)** Patient B60 demonstrated an enrichment of plasma DNA fragments at 160 bps whereas B49 had plasma DNA fragments at about 315 bps in addition. **(b)** Corresponding plasma-Seq profiles of B60 and B49. **(c)** Copy number patterns established from a panel of patients with metastatic breast cancer. Depicted are recurrent aberrations (upper panel gains, lower panel losses), the Y-axis represents relative abundance of respective aberrations of unbalanced copy number profiles. Data from patients with balanced copy number profiles were not included. These analyses revealed losses of 1p, 8p, 13q, and 16q, and gains of 1q, 8q, 16p, and 17q.

In a further 71 plasma probes derived from 57 patients with metastasized breast cancer we analyzed the plasma DNA size distribution and investigated the presence of copy number alterations. Characteristics of these patients, that is histological features of the primary and the localization of the metastases are detailed in Table S2 in Additional file 5. Two exemplary cases are illustrated in Figure 4a-b. Patient B60 had only an enrichment of DNA fragments in the range of approximately 160 bps (Figure 4a) and showed a balanced profile (Figure 4b), whereas patient B49 had an enrichment of DNA fragments at approximately 310 bps in addition (Figure 4a) and multiple copy number changes in the plasma-Seq analysis (Figure 4b). The presence of such longer DNA fragments appeared to be indeed a good indicator for an increased mutant AF fraction. We found copy number changes in 78.1% (25/32) of plasma samples with a biphasic (that is enrichment of fragments at approximately 160 bps and approximately 310 bps) plasma DNA size distribution but in only 7.7% (3/39) with a monophasic (that is only enrichment of approximately 160 bps fragments) plasma DNA size distribution (*P* <0.0001; chi-squared test). We analyzed the copy number patterns (Figure 4c) and found that these corresponded to those frequently observed in breast cancer [40], such as losses of 1p, 8p, 13q, and 16q, and gains of 1q, 8q, 16p, and 17q, demonstrating the specificity of our approach. We also tested whether the occurrence of copy number changes was correlated with any clinical characteristics of the patients or features of the tumor and found a statistical significant correlation with the presence of liver metastases (*P* = 0.002; chi-square test) but not for any other parameter. Overall, this suggests that patients with metastatic breast cancer may have highly variable AFs of mutant DNA in their circulation. This is in line with a recent study, which also reported that the concentration of ctDNA varied even among patients with the same tumor type [23].

The observed different patterns of mutant AFs in the plasma of advanced stage patients, as illustrated in Figure 4, suggest that the mechanism of ctDNA release may not be the same in all patients. The lengths of DNA fragments provide a glimpse into the mechanisms underlying ctDNA release into the circulation. Macrophages engulfing apoptotic or necrotic cells may release digested DNA into the circulation. As this process involves both neoplastic cells and surrounding stromal and inflammatory cells, the released DNA is a mixture of wild-type and mutant sequences. Plasma DNA fragments within the size range of 85 to 230 bps likely reflect enzymatic processing and release of DNA from apoptotic cells, because the length of these fragments corresponds to the DNA wrapped around a nucleosome (approximately 142 bps) plus a linker (approximately 20 bps) [37],[41]. Accordingly, the longer DNA fragments likely represent di- and trinucleosomal DNA. Hence, their presence suggests that in a subset of patients with cancer phagocytosis and subsequent release of the digested DNA into the circulation may be affected. Other contributing factors could consist of shorter degradation times or saturation of DNA degradation mechanisms due to high amounts of DNA.

## Conclusions

Clinical response evaluation of patients with cancer is often not accurate. Therefore, the use of ctDNA has been proposed as a biomarker for monitoring tumor burden and treatment response. Indeed, several studies have suggested that ctDNA analysis is an effective indicator of tumor load, allowing more accurate monitoring of tumor dynamics [10]-[13],[21],[37]. In breast cancer, a recent study has provided evidence that ctDNA levels had a greater dynamic range and greater correlation with changes in tumor burden than CA 15-3 or CTCs [13].

However, our study indicates that the biology of CTC and ctDNA release into the circulation is yet poorly understood, because we show that CTC numbers and ctDNA AFs may yield discrepant results. High-resolution plasma DNA fragment sizing suggested that differences in phagocytosis and DNA degradation mechanisms may contribute to variant ctDNA levels. Hence, the future use of both CTCs and ctDNA as liquid biopsy to monitor tumor disease will depend on an improved knowledge about mechanisms of CTC and ctDNA biology in cancer patients receiving systemic therapy.

## Authors' contributions

MH, MA, PU, EH, JBG, and MRS planed the project and the experimental design. EP, IL, and GP obtained, analyzed and interpreted the clinical data. SL performed all histopathological analyses. CG, SR, OM, and KP identified and isolated the CTCs and were involved in the initial amplification of the CTC DNA and the interpretation of all CTC and genomic data. MH, MA, PU, and EH performed the sequencing, deep-sequencing, next-generation sequencing and analysis of sequencing data. JBG and MRS wrote the manuscript. All authors read, revised critically for intellectual content, and approved the final manuscript. All authors agreed with the accuracy and integrity of any part of the work.

## Additional files

## Electronic supplementary material

Additional file 1: Table S1.: Summary of genes (that is *PCDH20*, *OR4X1*, *ALK*, *DNPEP*, *SH3TC2*, *DDR2*, *MLL3*, and *PIK3CA*) identified by exome sequencing, which were used to establish the frequency in the tumor lesions, lymph nodes, CTCs, and plasma DNA, and their frequency - as established by deep sequencing in the various samples. (XLS 35 KB)

Additional file 2: Treatment details regarding the index patient.(DOCX 14 KB)

Additional file 3: Figure S1.: Copy number profiles obtained by whole-genome sequencing, in each panel shows the X-axis the chromosome, the Y-axis indicates log_2_-ratios. **(a)** Tumor lesions A, C, and D. **(b)** Lymph node metastases LN15, LN17, and LNA. **(c)** Profiles from a single CTC, two 10 CTCs pools (10x pool1 and 10x pool2), and a pool of 50 CTCs (50x pool). (TIFF 575 KB)

Additional file 4: Figure S2.: Representative array-CGH profiles of single CTCs or pools of several CTCs. **(a)** Array-CGH profiles of CTCs from the first blood collection. The two top panels each show the profile of a single CTC, the third and the fourth panel depict profiles of pools of 10 or 50 CTCs, respectively. **(b)** Single-cell CTC array-CGH profiles of the second (first and second panel) and third (third panel) blood collection. (ZIP 2 MB)

Additional file 5: Table S2.: Summary of the histological features of the primary and the localization of the metastases of 57 patients with metastatic breast cancer. (XLSX 13 KB)

Below are the links to the authors’ original submitted files for images.

Authors’ original file for figure 1

Authors’ original file for figure 2

Authors’ original file for figure 3

Authors’ original file for figure 4

## Abbreviations

AF: allele fraction
BWA: Burrows-Wheeler algorithm
CA 15-3: cancer antigen 15-3
cfDNA: cell-free DNA
COSMIC: Catalogue of Somatic Mutations in Cancer
CTCs: circulating tumor cells
ctDNA: circulating tumor DNA
ER: estrogen receptor
GREVE: Genomic Recurrent Event ViEwer
PR: progesterone receptor
SNPs: single nucleotide polymorphisms
SNVs: single nucleotide variants
WGA: whole-genome amplification
