# Supplementary material for: The dynamic range of circulating tumor DNA in metastatic breast cancer
Source: Breast Cancer Res. 2014 Aug 9;16:421. doi: 10.1186/s13058-014-0421-y (PMC4303230; doi:10.1186/s13058-014-0421-y)
Supplement: Supplementary file 2 — Additional file 2: Treatment details regarding the index patient.(DOCX 14 KB) [file 13058_2014_421_MOESM2_ESM.docx]

**Additional_file_2 as DOC**
**Additional file 2** Treatment details regarding the index patient. Twenty-nine months after initial diagnosis, bone metastases and a thrombocytopenia (59,000/mm^3^) were noted (Figure 1), resulting in a change of therapy. The patient denied chemotherapy. Thus she received Fulvestrant, a selective estrogen receptor down-regulator, due to her postmenopausal status after chemotherapy. One month later, denosumab, a monoclonal antibody for the treatment of bone metastases, was added. At month 31 of follow up, several liver metastases were noted. Antihormonal therapy was changed to Anastrozole, an aromatase-inhibitor. Palliative chemotherapy with anthracyclines and/or taxanes was considered again. However, the patient again refused such treatment. At this time we obtained our first blood sample (Figure 1), which yielded the exceptional number of more than 100,000 CTCs with the CellSearch system. Four days after the first blood collection, the patient received bevacizumab, an angiogenesis inhibitor (15 mg/kg), although there was no proof of efficacy without chemotherapy at this time. Four days later, we received the second blood sample (Figure 1), which confirmed the presence of more than 100,000 CTCs. However, the size of liver metastases (diameter of 2.3 cm) increased and ascites was newly diagnosed. In addition, the physical condition of the patient (Karnofsky 60) worsened significantly over time. This resulted in switching the antihormonal treatment to Exemestane. About one month later, we obtained the 3^rd^ blood sample (Figure 1), which revealed a CTC number of ~50,000. Further clinical signs of subileus were noted and the patient died about 9 weeks after our last blood collection.
