# Supplementary figures and images for: The dynamic range of circulating tumor DNA in metastatic breast cancer
Source: Breast Cancer Res. 2014 Aug 9;16:421. doi: 10.1186/s13058-014-0421-y (PMC4303230; doi:10.1186/s13058-014-0421-y)

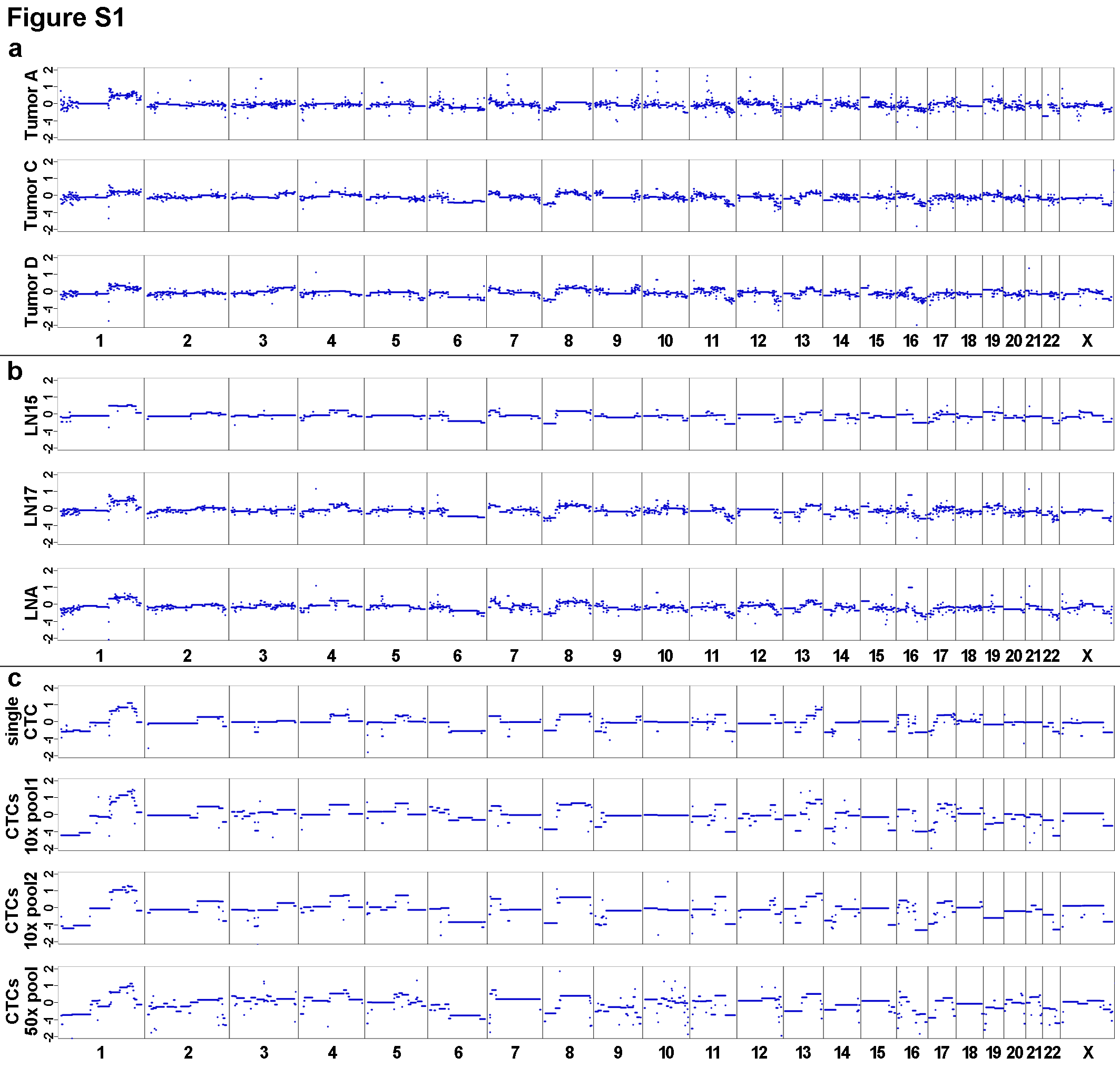

Supplement: Supplementary file 3 — Additional file 3: Figure S1.: Copy number profiles obtained by whole-genome sequencing, in each panel shows the X-axis the chromosome, the Y-axis indicates log2-ratios. (a) Tumor lesions A, C, and D. (b) Lymph node metastases LN15, LN17, and LNA. (c) Profiles from a single CTC, two 10 CTCs pools (10x pool1 and 10x pool2), and a pool of 50 CTCs (50x pool). (TIFF 575 KB) [file 13058_2014_421_MOESM3_ESM.tiff]

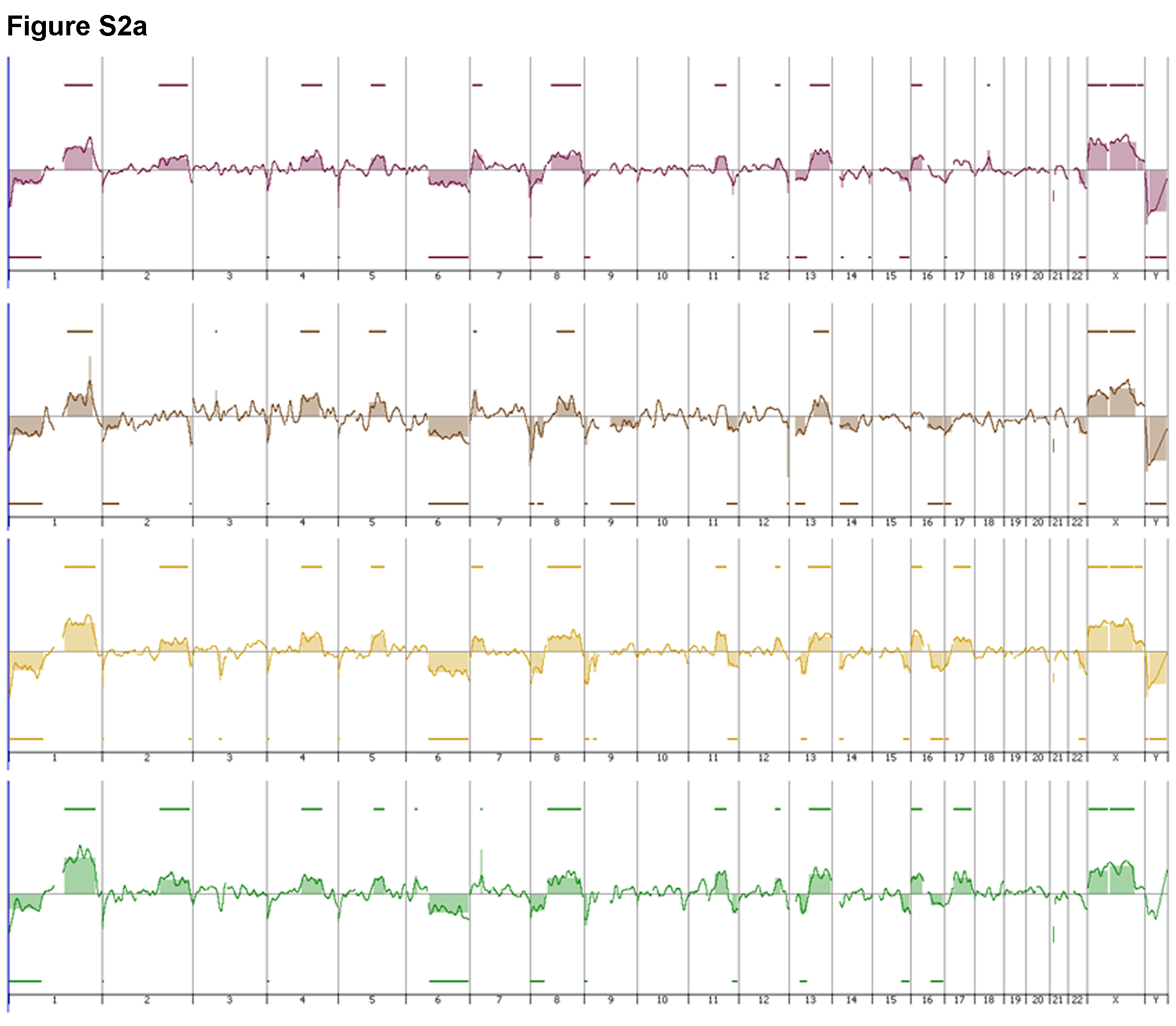

Supplement: Supplementary file 4 — Additional file 4: Figure S2.: Representative array-CGH profiles of single CTCs or pools of several CTCs. (a) Array-CGH profiles of CTCs from the first blood collection. The two top panels each show the profile of a single CTC, the third and the fourth panel depict profiles of pools of 10 or 50 CTCs, respectively. (b) Single-cell CTC array-CGH profiles of the second (first and second panel) and third (third panel) blood collection. (ZIP 2 MB) [file 13058_2014_421_MOESM4_ESM.zip › 7262059081176956_add3.tiff]

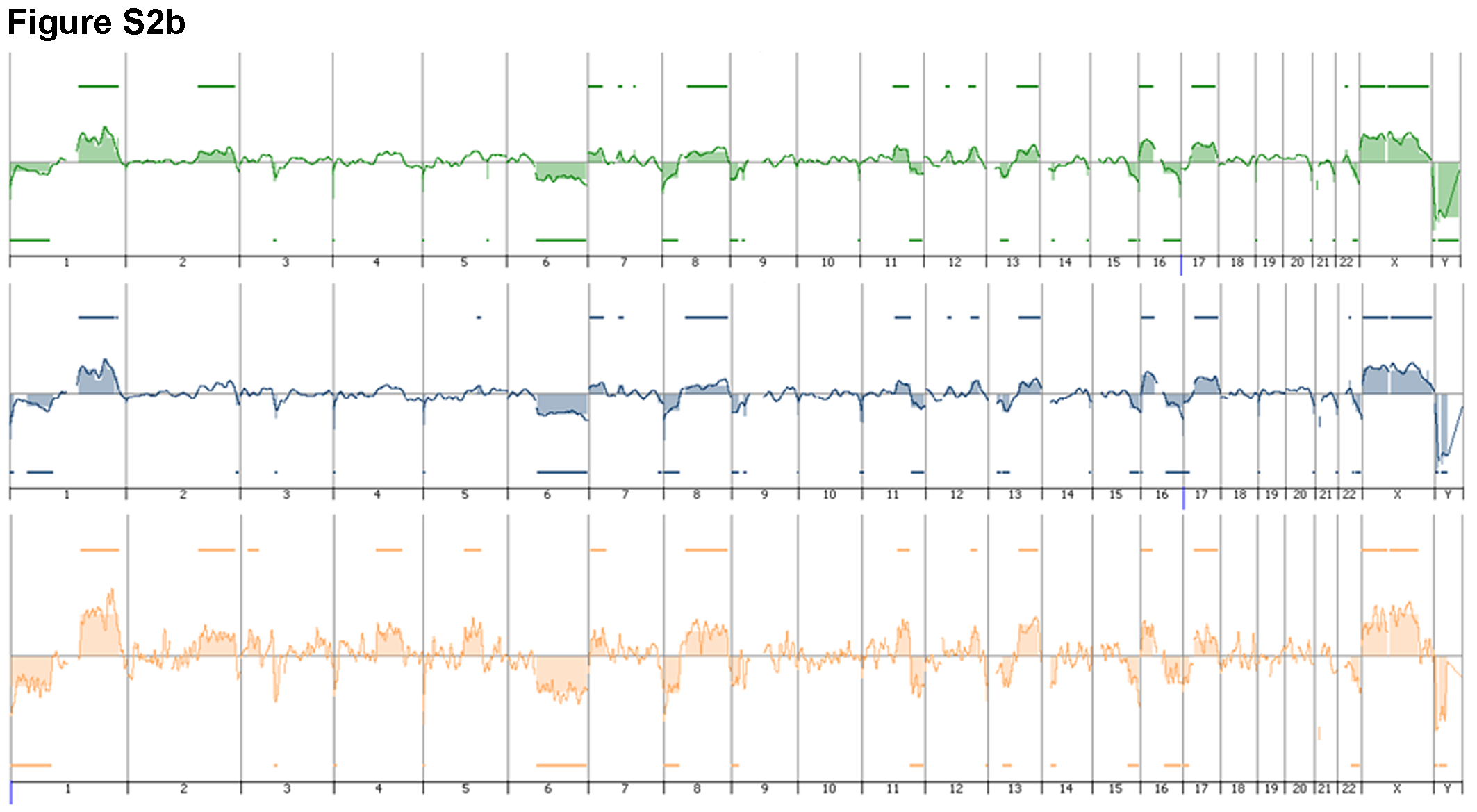

Supplement: Supplementary file 4 — Additional file 4: Figure S2.: Representative array-CGH profiles of single CTCs or pools of several CTCs. (a) Array-CGH profiles of CTCs from the first blood collection. The two top panels each show the profile of a single CTC, the third and the fourth panel depict profiles of pools of 10 or 50 CTCs, respectively. (b) Single-cell CTC array-CGH profiles of the second (first and second panel) and third (third panel) blood collection. (ZIP 2 MB) [file 13058_2014_421_MOESM4_ESM.zip › 7262059081176956_add4.tiff]

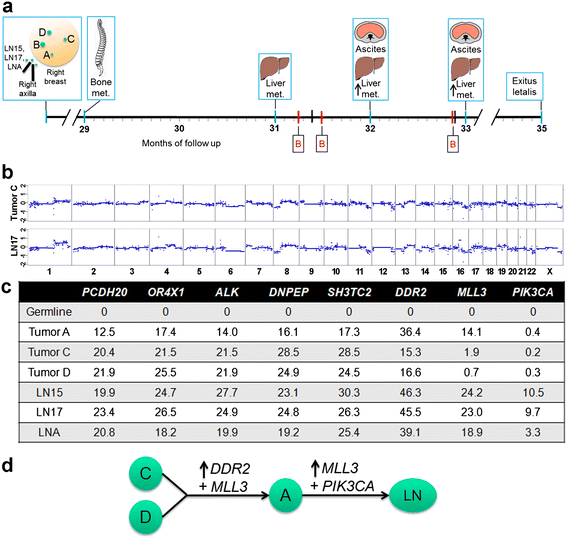

Supplement: Supplementary file 6 — Authors’ original file for figure 1 [file 13058_2014_421_MOESM6_ESM.gif]

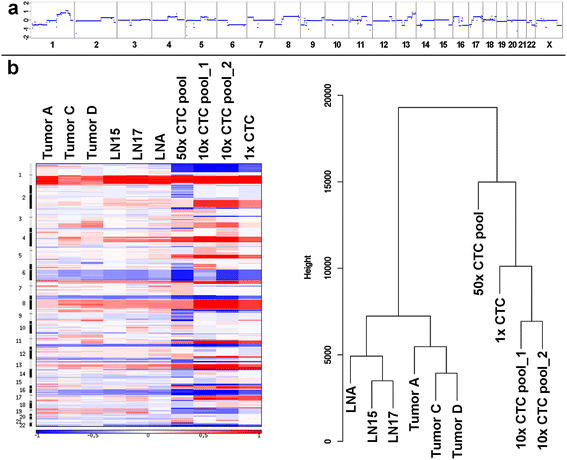

Supplement: Supplementary file 7 — Authors’ original file for figure 2 [file 13058_2014_421_MOESM7_ESM.gif]

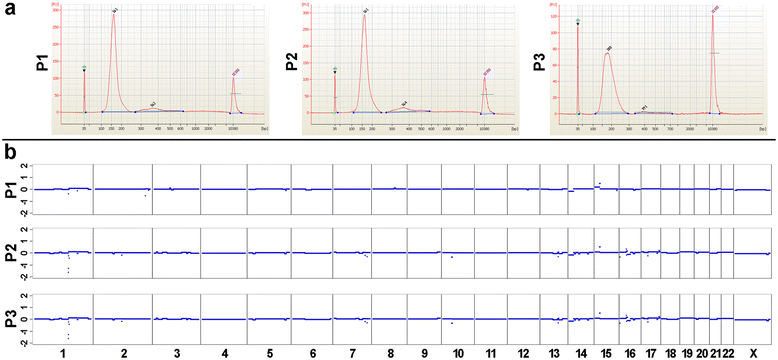

Supplement: Supplementary file 8 — Authors’ original file for figure 3 [file 13058_2014_421_MOESM8_ESM.gif]

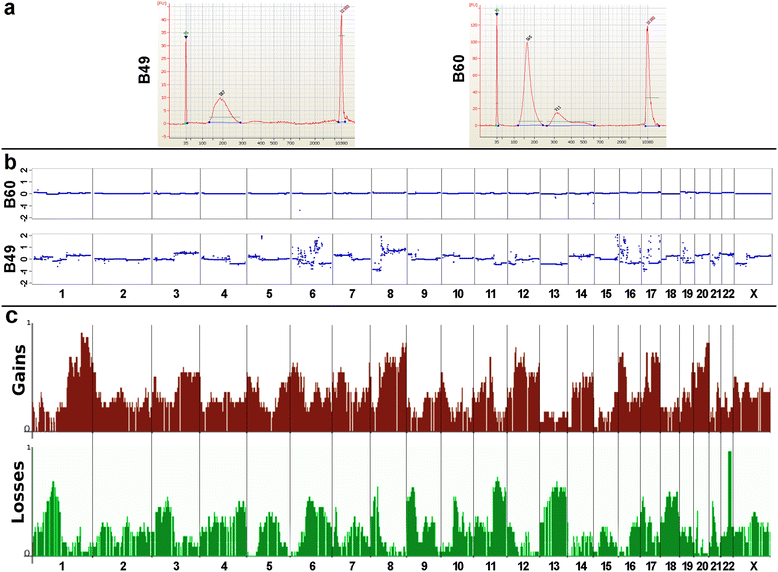

Supplement: Supplementary file 9 — Authors’ original file for figure 4 [file 13058_2014_421_MOESM9_ESM.gif]
